# Telocytes in the urinary system

**DOI:** 10.1186/1479-5876-10-188

**Published:** 2012-09-10

**Authors:** Yonghua Zheng, Tongyu Zhu, Miao Lin, Duojiao Wu, Xiangdong Wang

**Affiliations:** 1 Department of Pulmonary Medicine, Fudan University, Zhongshan Hospital, No.180, Fenglin Road, Shanghai 200032, China; 2 Biomedical Research Center, Fudan University, Zhongshan Hospital, No.180, Fenglin Road, Shanghai 200032, China; 3 Department of Urology Surgery, Fudan University, Zhongshan Hospital, No.180, Fenglin Road, Shanghai 200032, China; 4 Key Lab of Shanghai Organ Transplantation, Fudan University Zhongshan Hospital, No.180, Fenglin Road, Shanghai 200032, China

**Keywords:** Telocytes, Kidney, Ureter, Urinary bladder

## Abstract

**Background:**

Telocytes, a new type of interstitial cells, have been identified in many organs in mammals. The present studies aimed at investigating the ultrastructure, distribution and interactions of telocytes with surrounding cells in the urinary system of rats, to confirm the existence of telocytes in kidneys, ureter and urinary bladder.

**Methods:**

Samples of kidney, ureter, or urinary bladder were harvested for the ultrastructure by the electron microscope. The primary culture of telocytes was performed to investigate the dynamic alterations.

**Results:**

Telocytes mainly located in the sub-capsular space of kidney, or between smooth muscle bundles and in the lamina propria of ureter and urinary bladder. Telocytes established numerous contacts with macrophages in the sub-capsular space of kidney, or with smooth muscle cells, nerve endings as well as blood capillaries in the ureter and urinary bladder. The complete morphology of telocytes with telopodes was observed clearly through the primary cell culture from the kidney tissues of rats.

**Conclusions:**

Our data evidenced the existence of telocytes in the urinary system, which may contribute to the tissue reparation and regeneration.

## Background

There is increasing evidence of telocytes as a new type of interstitial cells recently, of which the most focused on the location and morphologic characteristics. Telocytes are characterized by specific ultrastructural features of telopodes thin fibrillar-like thin segments (podomeres) and dilated, beads-like thick regions (podoms) [1-3]. Telopodes contain a large number of mitochondria, endoplasmic reticulum and caveolae, and could secret exsomes. Telocytes per se or with others are connected by telpodes and the form of networks. Cismasiu VB et al. [4] found that miR-193 was highly expressed in telocytes rather than other stromal cells and suggested that telocytes could be specialized and characterized by the expression of miR-193, if the morphologies could be clarified. Telocytes were also identified in stem cell niches and connected with precursor stem cells in the heart, lung, skeletal muscle or skin [5-9]. It was indicated that telocytes might be associated with the regeneration and reparation of injured tissues and organs, through the signal transduction of telopodes and secretion of exsomes.

Telocytes were detected in a number of tissues/organs in mammals, e.g. heart [10-16], blood vessels [17], placenta [18], exocrine pancreas [19], intestine [20-22], trachea [23,24], lungs [7,23], pleura [25], skeletal muscle [8,26], uterus and fallopian tube [27,28], urinary tract [29], skin [9,30], endometrium [31], parotid glands [32], or meninges and choroid plexus [33]. There is still a lack of telocytes in the kidney and urinary bladder, even though telocytes were seen in the upper lamina propria of the human urinary tract [29]. The present study aimed to investigate the existence, characteristics, and distribution of telocytes in the kidney and urinary bladder and observe dynamic alterations of isolated and cultured telocytes from the kidney.

## Methods

### Animals

Three Sprague–Dawley rats were obtained and maintained from the animal research center of Fudan University, Shanghai, China. Rats, male, 8-week-old, weighing 200-250 g, were housed in a local facility for laboratory animal care and held, fed *ad libitum*, according to the local ethical guidelines. The study was approved by the Ethic Committee for Animal Care and Use, Fudan University, and performed according to accepted international standards.

### Transmission electron microscopy

For ultrastructural analysis, tissue samples of kidney, ureter and urinary bladder were cut into small pieces about 1 mm^3^ within 1 min after being excised from rat body and immediately immersed in a solution of 4% glutaraldehyde (pH 7.3, 4°C). Fixed samples were washed in phosphate buffer, and were post-fixed in 1% osmium tetroxide (Polysciences Inc. Warrington, USA) for 1 hr. Samples were then rinsed extensively in 0.1 M cacodylate buffer. Following several rinses in 0.1 M cacodylate buffer, samples were dehydrated in a graded series of ethanol and were embedded in Epon 812 resin (Ted Pella Inc. California, USA). The embedded samples were dried by heat with serial temperatures (37°C for overnight, 45°C for 12 hrs and 60°C for 48 hrs). Then sections of 50 nm were cut with a Leica Ultracut UCT ultramicrotome (Leica Microsystems Inc, LKB-II, Germany), stained with 3% solution of uranyl acetate and lead citrate, and mounted on formvar coated 50 mesh grids. Digital pictures (2048 × 2048 pixels, 4 MB, and uncompressed grayscale Tiff files) were obtained using a high resolution digital camera MegaViewIII (SIS®) connected to the TEM, and observed at an acceleration voltage of 80 kV, in JEOL JEM-1230 (Japan) electron microscope.

### Isolation and primary cell culture of renal telocytes

Rats were euthanized with pentobarbital sodium (1%, 0.4 ml/100 g) by peritoneal injection. The kidneys were cut and harvested under sterile conditions and collected into sterile tubes containing Dulbecco’s Modified Eagle’s Medium (DMEM, Gibco, New York, USA), supplemented with 100 UI/ml penicillin and 0.1 mg/ml streptomycin (Sigma Chemical, St. Louis, MO, USA), and the samples were brought to the cell culture room immediately. Under the sterile conditions, the kidney samples were rinsed with sterile DMEM and minced into fragments about 1 mm^3^, which were then incubated at 37°C for 4 hrs on an orbital shaker, with 10 mg/ml type II collagenase (Sigma, St. Louis, MO, USA) in PBS without Ca^2+^ and Mg^2+^. Dispersed cells were separated from non-digested tissue by the filtration through a 40 μm diameter cell strainer (BD Falcon, Franklin, New Jersey, USA), collected by centrifugation at 2000 rmp, 5 min, and were resuspended in DMEM, supplemented with 10% fetal calf serum (Gibco, New York, USA), 100 UI/ml penicillin and 0.1 mg/ml streptomycin. Cell density was counted in a haemocytometer and viability was assessed using the Trypan blue dye exclusion test. Cells were distributed in 25 cm^2^ plastic culture flasks, at a density of 1 × 10^5^ cells/cm^2^, and maintained in a humidified incubator at 37°C with 5% CO_2_ until becoming semi-confluent (usually 4 days after plating). Culture medium was changed every 48 hrs. Cultured cells were examined by phase contrast microscope, under an inverted Olympus phase contrast microscope (1×51), and the typical telocytes were photographed.

## Results

### Transmission electron microscopy

**Figure 1:**
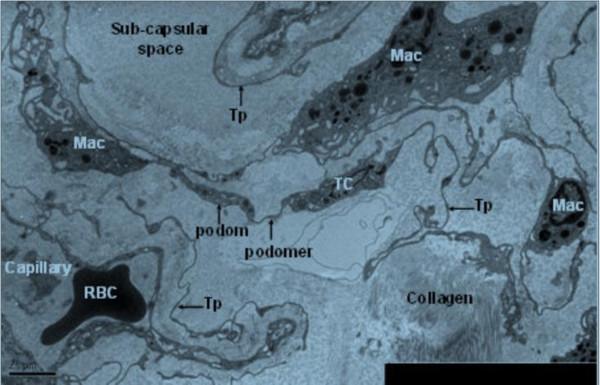
**TEM of rat kidney. **A Telocyte with its long telopode is visible under sub-capsular space of kidney, and it is near the capillary. Noting the striking labyrinthine network formed by telopode. Telopode is very long with alternation of podom and podomer. Telocyte connects with macrophages by telopode. (TC: telocyte; Tp: telopode; Mac: macrophage; RBC: red blood cell). Magnification is 5000×, and the scale bar is 2 μm.

In kidney, telocytes with long telopode could be seen clearly in sub-capsular space, and they connected with macrophages through telopode. Telopodes were detected as discontinuous segments with alternation of podom and podomer due to the three dimensional extension of telopode. Telocytes connected with telopodes and existed near the blood vessels, and there were network structures or labyrinthine structure, between telopodes (Figure 1).

**Figure 2:**
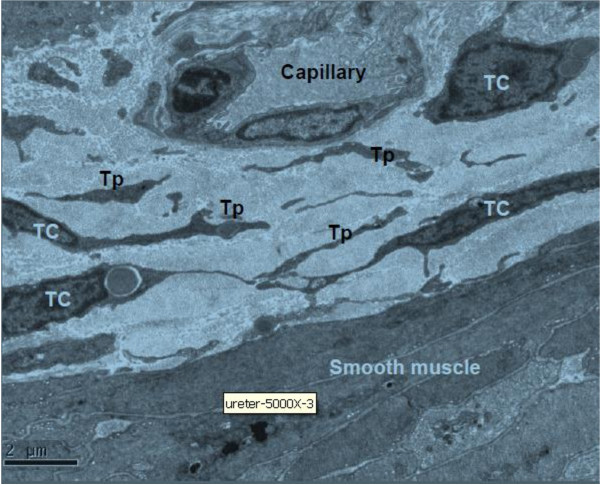
**TEM of rat ureter. **Telocyte and telopode exist in lamina propria of ureter, and they connect with capillaries and smooth muscles. Four telocytes are showed. (TC: telocyte; Tp: telopode). Magnification is × 5000, and the scale bar is 2 μm.

**Figure 3:**
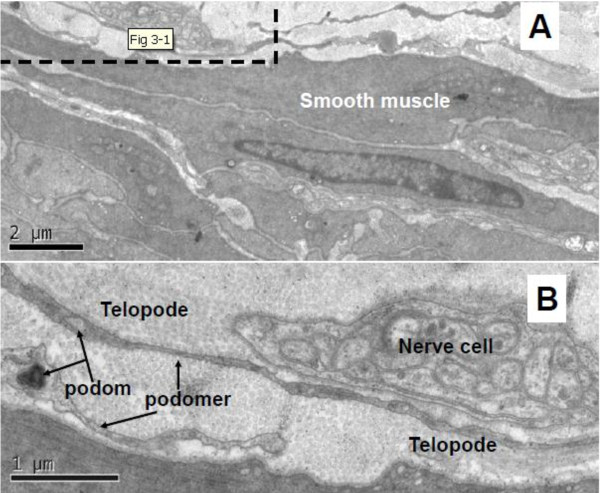
**TEM of rat ureter. **A shows telopode exist in lamina propria of ureter, and it connects with never ending. Podom and podomer are showed clearly. Magnification is × 5000, and the scale bar is 2 μm. **B **is the enlargement of black dotted frame in **A**. Magnification is × 15000, and the scale bar is 1 μm.

**Figure 4:**
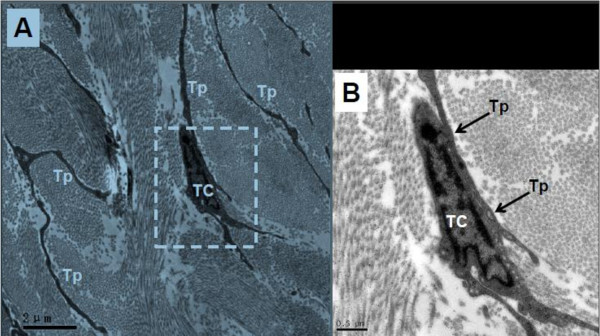
**TEM of rat ureter. A **shows typical telopodes (Tp) and telocytes (TC) in the ureter, and telocytes connect with telopodes from other telocytes. **B** is the enlargement of white dotted frame in A, and the details about the connection between telcoytes (TC) and telopode (Tp, black arrow) are showed clearly. Magnification of A is 5000 ×, and the scale bar is 2 μm. Magnification of B is 20000 ×, and the scale bar is 0.5 μm.

**Figure 5:**
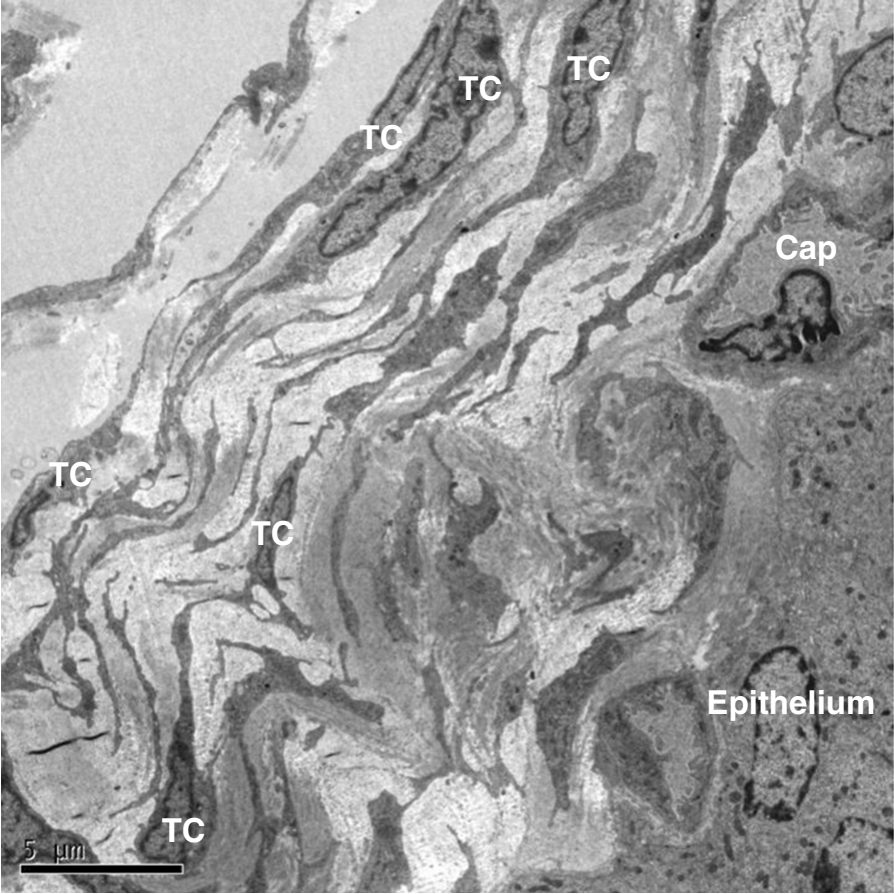
**TEM of rat urinary bladder.**At least 6 telocytes with their extensive telopodes are visible in the interstitium of epithelium, near the capillaries, and they form a labyrinthine structure. (TC: telocytes; Cap: capillary). Magnification is 3000×, and the scale bar is 5 μm.

In ureter and urinary bladder, telocytes mainly exist in between smooth muscle bundles, where in the lamina propria, and their telopodes made a network which appeared like labyrinthine structure and were also detected as discontinuous segments with alternation of podom and podomer. The nucleus of telocytes appeared in irregular shapes and contained clusters of heterochromatin attached to the nuclear envelope. Telocytes are connected with smooth muscles, capillaries and never endings (Figure 2, Figure 3, Figure 5) by their long telopodes. Telocytes could connect with each other closely through their telopodes, as shows in Figure 4.

### Primary cell culture of telocytes

**Figure 6:**
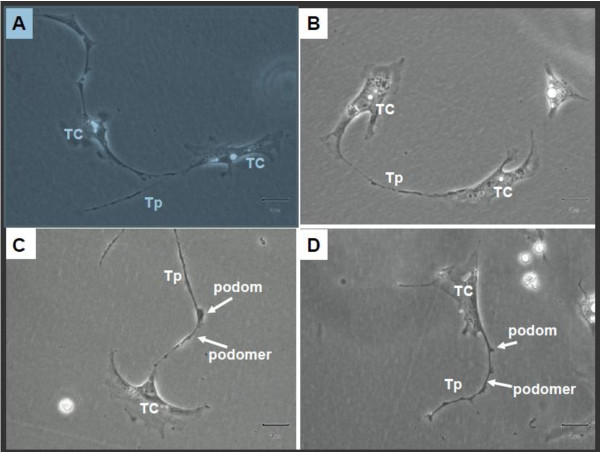
**Telocytes from rat sub-capsular space of kidney. **Primary culture, 5 days. There are two telocytes with typical Tp in **A**/**B**, and they connect together. Single telocyte with typical telopode (alternation with podom and podomer) could be seen in **C**/**D **clearly. (TC: telocytes; Tp: telopode). Magnification is 200× , and the scale bar is 3 μm.

In our study, telocytes were cultured and identified at the third day before reaching confluence. Telocytes with long telopodes and uneven podoms and podomers were observed from the third and fourth days on (Figure 6). The length seemed to be related with the duration of culture in the short term of cell growth, although the mechanism and long-term situation remain unclear. We found that telopodes could connect with each other frequently by either side-to-side of telopodes (Figure 6A) or end-to-end of telopodes (Figure 6B) and occasionally by end-to-side. This implies that the telopodes might deliver the signal either by the pass-by during cellular functioning or special and unique communication between telocytes. The shape of telocytes changed at different time schedules and telopodes changed from wide to thin. Telopodes extended and moved, leading to alternation of thin segments (podomers) and dilations (podoms) (Figure 6C and D).

## Discussion

The special cells we identified in the urinary system of rats were in line with the diagnostic criteria of telocytes, which was defined by Popescu et al. in 2010[1]. As it was showed in the results, telocytes were identified in the interstitial spaces of urinary system in rats, and this added new evidence for the existence of telcoytes in the mammals. Based on our own study, about 2–3 telopodes were observed on a single section, dependent upon the site and angle of section, since it was hardly to see 3 dimensional convolutions of telopode at the full length in a section. The lengths of telopodes were tens to hundreds of micrometers, as measured on EM images [1], while it was detected during cell cultures in the present study. The length seemed to be related with the duration of culture in the short term of cell growth, although the mechanism and long-term situation remain unclear. We found that telopodes could connect with each other frequently by either end-to-end or side-to-side and occasionally by end-to-side. This implies that telopodes might deliver the signal either by the pass during cellular functioning or by special and unique communication between telocytes, as described by Suciu L et al. [33]. Because the morphology and structure of the cells are always associated with their functions, there must be many other important functions for telocytes.

According to the published results, some potential roles of telocytes could be summarized. Popescu et al. [1] suggested that telocytes were involved in intercellular signaling, for their three dimensional network of telopodes and their strategic position with other cells, blood capillary and nerve ending. Telcoytes also play roles in vascular system, nervous system, immune system, interstitium and stem cells/progenitors [6]. Laura Suciu et al. [18] suggested that telcoytes might be involved in the angiogenesis, since they found telocytes expressed CD34 and VEGF. Manole CG et al. [34] found that telocytes involved in neo-angiogenesis in the experimental acute myocardial infarction. Telocytes might be involved in tissue regeneration and reparation [9,26].

The function of telocytes in kidney might be involved in the reparation of injured tissues and immune reflections during diseases such as acute kidney injury, renal failure and renal fibrosis. It is supported by our findings that telocytes and telopodes were located in close vicinity of blood capillaries and connected with macrophages in the sub-capsular space of kidney. The existence of telopodes and the 3 dimentional network implies that telocytes may play an important role in the intercellular communication and regulation [3]. The function of telocytes in ureter and urinary bladder might be involved in the reparation of injured tissues during diseases, like in the skeletal muscles [26]. It is supported by our findings that telocytes and telopodes were located in close vicinity of blood capillaries, nerve endings and smooth muscles. However, there is a great need to explore telocyte-specific biomarker to clarify the cell in the functional aspect and in an easier way, network biomarkers to understand more about the interaction between proteins, genes, and signal pathways, and dynamic network works to define and predict time-dependent telocyte function and morphological features [35-38]. It will be more challenging to translate those experimental knowledge and evidences into the understanding of diseases and application of clinical diagnoses and therapies in the aspects of clinical and translational medicine [34,39-41].

In conclusion, the present study provided ultrastructural evidence for the existence of telocytes in kidney, ureter and urinary bladder. Telopodes connect each other by end-to-end or side-to-side both in the tissue and cell culture. There are still further needs to explore potential bio-functions of telocytes in certain pathological conditions in urinary system and mechanism of interaction between telocytes and other cells.

## Competing interests

The authors declare that they have no competing interests.

## Authors’ contributions

YHZ performed studies, result calculation, images processing, and the writing of manuscript. TYZ participated in the design of the study and images processing. ML performed the study. DJW participated in the design of the study. XDW participated in the design, result calculation and analysis, and helped to draft the manuscript. All authors read and approve the final manuscript.
